# Multi-Phase Fusion for Pedestrian Localization Using Mass-Market GNSS and MEMS Sensors

**DOI:** 10.3390/s23073624

**Published:** 2023-03-30

**Authors:** Qiang Liu, Rendong Ying, Zhendong Dai, Yuze Wang, Jiuchao Qian, Peilin Liu

**Affiliations:** School of Electronic Information and Electrical Engineering, Shanghai Jiao Tong University, Shanghai 200240, China; lugialiu@sjtu.edu.cn (Q.L.);

**Keywords:** PDR, GNSS, fusion positioning, multipath mitigation, smartphone-grade sensors

## Abstract

Precise pedestrian positioning based on smartphone-grade sensors has been a research hotspot for several years. Due to the poor performance of the mass-market Micro-Electro-Mechanical Systems (MEMS) Magnetic, Angular Rate, and Gravity (MARG) sensors, the standalone pedestrian dead reckoning (PDR) module cannot avoid long-time heading drift, which leads to the failure of the entire positioning system. In outdoor scenes, the Global Navigation Satellite System (GNSS) is one of the most popular positioning systems, and smartphone users can use it to acquire absolute coordinates. However, the smartphone’s ultra-low-cost GNSS module is limited by some components such as the antenna, and so it is susceptible to serious interference from the multipath effect, which is a main error source of smartphone-based GNSS positioning. In this paper, we propose a multi-phase GNSS/PDR fusion framework to overcome the limitations of standalone modules. The first phase is to build a pseudorange double-difference based on smartphone and reference stations, the second phase proposes a novel multipath mitigation method based on multipath partial parameters estimation (MPPE) and a Double-Difference Code-Minus-Carrier (DDCMC) filter, and the third phase is to propose the joint stride lengths and heading estimations of the two standalone modules, to reduce the long-time drift and noise. The experimental results demonstrate that the proposed multipath error estimation can effectively suppress the double-difference multipath error exceeding 4 m, and compared to other methods, our fusion method achieves a minimum error RMSE of 1.63 m in positioning accuracy, and a minimum error RMSE of 4.71 m in long-time robustness for 20 min of continuous walking.

## 1. Introduction

The Location Based Service (LBS) is one of the daily applications for smartphones, and pedestrian positioning is the technical fundamental of LBS. Pedestrians can use several smartphone sensors to determine their location outdoors; for example, they can use GNSS to obtain latitude and longitude, or MARG sensors to achieve relative positioning. However, the current mass-market GNSS is limited by its ultra-low-cost hardware, such as antenna, RF front-end, etc., and the quality of its observation is at a huge disadvantage compared to professional receivers [1,2]. Moreover, GNSS is also subjected to multipath interference in a complex pedestrian scene such as a crowded city street, which leads to the severe degradation of GNSS single-point positioning accuracy, usually, 3 to 10 m [3,4]. Similarly, smartphone-grade MEMS MARG faces the challenge of long-time drift, which is quite difficult to solve. Compared with professional devices, smartphone-grade sensors have great advantages in size, power consumption, and price; as a result, they enhance the user experience of LBS, but these advantages are built upon the sacrifice of performance. Therefore, outdoor users cannot obtain satisfactory positioning results by relying on GNSS or MARG alone. To combine the advantages of different sensors and to improve the overall performance, sensor fusion has received extensive attention from scholars [5,6,7].

MEMS MARG includes a 3-axis accelerometer, a 3-axis gyroscope, and a 3-axis magnetometer, where the first two are part of the Inertial Measurement Unit (IMU). Compared with industrial- or tactical-grade MEMS IMU, the smartphone-grade IMU has a huge performance gap [8,9]. Therefore, smartphone IMUs are not able to implement a Strap-down Inertial Navigation System (SINS), as with professional IMUs. SINS calculates the position, velocity, and attitude by integrating the observations of IMU, but the measurement error of the smartphone’s IMU is so large that it is amplified in the integration process, failing SINS in a very short time. However, in some applications, such as pedestrian navigation, MEMS IMU produces regular signals due to the characteristics of human walking, so PDR was invented [10,11,12]. PDR extracts the high-level behavior features from the raw data, and it avoids too many integration operations to effectively reduce the error drift, but the essence of PDR is still dead reckoning; this means that accumulated errors are still inevitable.

The embedded GNSS module in early smartphones was able to output only positioning coordinates, so scholars were unable to implement complex positioning algorithms based on a smartphone’s GNSS, such as Difference GNSS (DGNSS). Android OS has granted users access to GNSS raw measurements, which prompted research on the high precision measurements of the mass-market GNSS chipset to spring up [13,14]. Nowadays, the GNSS chipset can output both pseudorange and carrier phase observations, but the quality of the carrier phase is too poor to be used for ranging [15,16]. At present, smartphones mainly adopt the single-point positioning of pseudorange observation to calculate the receiver position, which is highly influenced by the multipath error and thermal noise, thus significantly reducing the positioning accuracy. Fortunately, scholars have two feasible ways to improve the accuracy of pseudorange positioning; one is to use the Continuously Operating Reference Station (CORS) network to obtain the reference station data and to construct double-difference positioning to eliminate most of the errors [17], and the other is to make better use of other GNSS observations, such as the carrier-to-noise-density ratio (CN_0_R).

The integrated navigation of GNSS and SINS is a very mature technology, and it has proven to be successful in many fields, which reflects the fact that GNSS and IMU are highly complementary [18,19]. Therefore, scholars associate to fuse GNSS and PDR, and to try to borrow the design idea of GNSS/SINS integrated navigation. Although GNSS/PDR fusion positioning can benefit from integrated navigation, it still faces many challenges: (1) When GNSS and PDR can only use ultra-low-cost sensors, how do we deal with their own measurement errors, such as GNSS multipath error? (2) The output data types of GNSS and PDR are quite different in terms of the coordinate form and output frequency, how should we unify them? (3) How do we design a fusion algorithm that minimizes the long-time drift of PDR? In this paper, we propose a multi-phase GNSS/PDR fusion framework based on smartphone-grade sensors. The first phase is to construct a Real-Time Difference (RTD) model between the smartphone and the reference station to replace traditional single-point positioning, the second phase is to propose a novel multipath error estimation method, and the last phase is to propose the joint stride length and heading estimation of the two standalone modules to improve the ability of the entire system to suppress long-time drift by designing a unique Kalman Filter (KF). The contributions of this paper are as follows:

- We adopt RTD instead of single-point GNSS in traditional fusion schemes, and propose a multipath mitigation algorithm that can be implemented on smartphones based on the MPPE and DDCMC filters, which exploits the CN_0_R and DDCMC observables;
- For the issue of different GNSS and PDR output formats, we design the coordinate transformation to convert GNSS data into a dead reckoning form, and the data synchronization to fix the results of PDR as periodic output;
- Before the conventional fusion filtering, we add the stride length and heading estimation modules to suppress the long-time drift of the MEMS sensors, which smooths the drift-free and noisy GNSS outputs with the drifted and low-noise PDR outputs.

The rest of this paper is organized as follows. Section 2 introduces some early related work. Section 3 presents the proposed fusion framework and positioning error analysis. Section 4, Section 5 and Section 6 give detailed descriptions of PDR, GNSS RTD, and the fusion system, respectively. Section 7 presents the setup and results of three different tests. Finally, Section 8 concludes our paper.

## 2. Related Work

PDR was initially used in those GNSS-denied environments, such as indoor positioning. In the last decade, a lot of literature [20,21,22] involving PDR has been published, and these previous works provided in-depth analysis and innovations, such as stride length estimation and heading estimation. Due to the fact that GNSS observation open access is a few years later than MEMS MARG integration into smartphones, early research on GNSS/PDR fusion commonly used professional- or vehicle-grade GNSS receivers, such as u-blox. Compared with bulky, high power consumption and expensive professional receivers, the miniaturized smartphone-grade GNSS chip has more advantages in new applications such as Internet of Things (IoT) and Unmanned Aerial Vehicle (UAV).

Positioning based on smartphone-grade GNSS can not only use pseudorange observations but it can also use carrier phase observations to achieve a higher accuracy, such as centimeter-level positioning, which has been widely explored in the GNSS community in recent years [23,24,25]. However, there are still many difficulties in the smartphone-grade carrier phase that have not yet been resolved, such as frequent cycle slips, tracking loss under weak signals, etc. Moreover, the characteristics of the carrier phase of different smartphones are different, requiring specific analysis, which is very complex. At present, carrier-based high-precision positioning is still in the exploration stage, and there is no mature solution. Carrier-based positioning also requires the resolution of integer ambiguity, and currently, the fixed time of smartphone-grade observations takes several minutes or even longer; the long fixed time cannot meet the need for pedestrian positioning because pedestrians cannot tolerate such a long waiting time. In addition, if the large multipath error in the smartphone’s pseudorange cannot be eliminated, the integer ambiguity estimation based on pseudorange and carrier phase combination cannot be promoted. Therefore, RTD positioning is adopted in this paper, which improves the accuracy compared with single-point positioning, and reduces the waiting time compared with carrier-based positioning.

According to the above analysis, RTD is suitable for pedestrian positioning, but smartphone-based RTD still suffers from severe multipath interference, partly caused by the application environment and partly caused by its ultra-low-cost antenna. A Planar Inverted-F Antenna (PIFA) is usually adopted in smartphones, and it is an omnidirectional, linearly polarized antenna. Therefore, a pedestrian will receive Non-Line-of-Sight (NLOS) signals from multiple directions, in addition to the Line-of-Sight (LOS) signal. Even worse, when the direction of the phone held by the pedestrian changes, that is, the antenna is not facing the zenith, then even the LOS signal will be blocked. These NLOS signals form constructive or destructive additions at the RF level, thus leading to unpredictable multipath errors in pseudorange observations. Conventional multipath mitigation methods include antenna arrays [26], longer pseudo-random code [27], multi-channel Delay Lock Loop [28], the statistical modeling of multipath electrical parameters [29], the 3D modeling of urban environments [30], etc. The above methods are highly demanding on hardware resources and power consumption, so that none of them can be applied to smartphones. In this paper, we consider exploiting the relationship between multiple GNSS observations [31,32], which can be implemented on smartphones at a lower cost, as detailed in Section 5.

Regarding GNSS/PDR fusion positioning. Hsu et al. [33] propose a framework of the fusion algorithm based on PDR and 3D map-aided GNSS in urban environments. They use a KF to process the inputs of the two subsystems, and the observations of the KF come from 3D map-aided GNSS, while the real-time outputs of PDR are used to update the state transition equation; this is because the trajectory of a pedestrian is very uncertain and is difficult to be described using the linear state transition equation. Lan et al. [34,35] use a state constraint KF to fuse PDR and GNSS, and their key algorithm is called the Error State Correction (ESC). Their analysis of PDR errors is very detailed and valuable because it is based on MEMS device errors. They use the headings derived from GNSS to assist with the heading estimation of the MEMS sensors, which improves the overall accuracy of the heading estimation, but the noise in the GNSS heading estimation has not been solved well, so that the initial stage of the pedestrian trajectory relies heavily on the GNSS. Basso et al. [36,37] propose a fusion method called Multi-Rate Extended Kalman Filter (MREKF), which is an event-triggered multi-rate size-varying KF. The data fusion is designed to exploit the real-time estimates of heading and stride length, provided by the PDR, with the position being obtained from GNSS. MREKF can work at each step with reduced information from the sensors, thus covering both the case of poor satellite coverage and the situation of different sampling rates for each source.

The main novelty of our proposed fusion method is the differentiated treatment of multipath errors and long-time drift. We consider the different characteristics of these two errors and think that it is inappropriate to fuse them directly with a common KF. In this paper, the multipath errors are first eliminated in the RTD subsystem, then the GNSS and PDR data are initially fused with the joint estimation to suppress the long-time drift, and finally, the filtering of the noise and residual errors is completed in the data fusion KF.

## 3. Fusion Framework and Error Analysis

### 3.1. Implementations of the Fusion Framework

**Figure 1:**
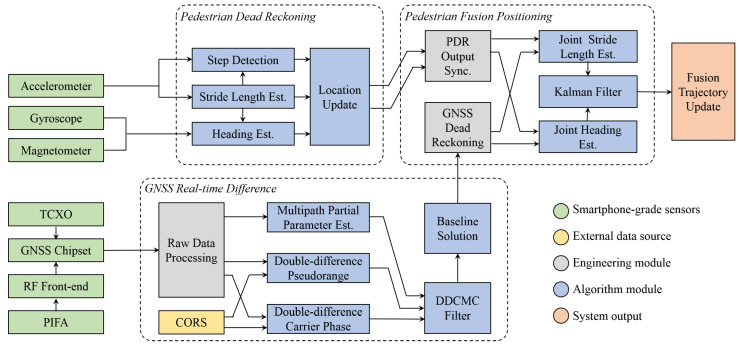
Block diagram of PDR and GNSS fusion framework based on smartphone-grade sensors.

The GNSS/PDR fusion framework proposed in this paper is shown in Figure 1, which mainly includes three parts: PDR module, GNSS RTD module, and fusion positioning module. A brief introduction to them is as follows:

- The inputs of PDR come from 9-axis MEMS MARG sensors, where the accelerometer data are used for step detection and stride length estimation, and the gyroscope and magnetometer data are used for heading estimation. Finally, the estimated stride length μ and heading θ are fed into the location update module to obtain the 2D coordinates $\left(x,y\right)$ of the pedestrian.
- GNSS RTD refers to the short-baseline pseudorange double-difference, and so, by receiving external CORS data to construct a double-difference model with the local data, most of the observation errors can be eliminated. Due to the poor performance of GNSS hardware, careful raw data processing is required before using them. MPPE and DDCMC filter are applied here to mitigate the multipath error, and the baseline solution can be obtained by solving the double-difference observation equation;
- Before fusing the GNSS and PDR data, their coordinate formats and timestamps need to be aligned, and these are performed in GNSS dead reckoning and PDR output synchronization. Then, the stride length and heading of the two subsystems are fed into their respective joint estimation modules. Finally, a KF is used to complete the fusion filtering, and the fusion trajectory update module outputs the positioning results.

### 3.2. Error Analysis of the Fusion System

In the GNSS/PDR fusion system, there are mainly three kinds of errors: (1) stride length and heading error in PDR: these two errors depend on the performance of the smartphone’s MEMS sensors; (2) double-difference pseudorange multipath error in RTD, this error is mainly related to smartphone’s antenna and operational environment; (3) random noise such as thermal noise in GNSS measurements.

#### 3.2.1. Cumulative Error of PDR

The stride length and heading errors of PDR can be modeled according to the characteristics of MEMS sensors; however, there are many MEMS error sources, so such an analysis is extremely complex [35]. To simplify the analysis, we observe the effect of stride length and heading errors on the final pedestrian trajectory. If the initial position is $\left(x_{0},y_{0}\right)$, then the kth position update is as follows:

(1) $$\left\{\begin{array}{ccc} \begin{array}{cc} x_{k} & =x_{0}+\sum_{i=1}^{k}\mu_{i}\sin\theta_{i}\\ y_{k} & =y_{0}+\sum_{i=1}^{k}\mu_{i}\cos\theta_{i} \end{array} & , & \forall k\in N^{+} \end{array}\right.$$

We can see that the stride length and heading errors of each step are accumulated continuously in the iterative process. If we know the stride length and heading errors at each step, we can deduce the influence of the heading and stride length errors on the final position from Equation (ER1), but this is still inconvenient. Therefore, we adopt an experimental method called the control point test, to replace the mathematical analysis, to observe the cumulative error of PDR. This test sets some control points (the coordinates have been calibrated) in advance, and the stride length and heading of each step can be fixed to a constant. Through the analysis of the real-time collected data, the characteristics of the stride length and heading errors can be observed. From Section 7, we can find that the errors of PDR increase with time, while the errors of GNSS are more similar to the zero-mean Gaussian noise.

#### 3.2.2. Influence of the GNSS Multipath on Observables

**Figure 2:**
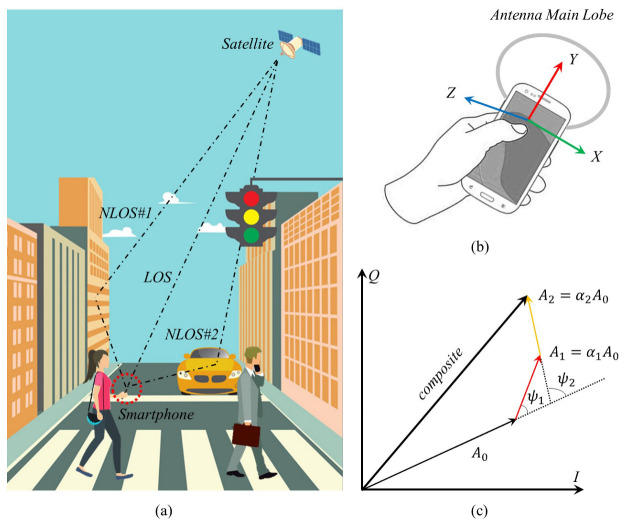
Multipath scene. (**a**) In urban streets, a pedestrian’s smartphone receives not only LOS from the satellite, but also NLOS♯1 reflected from buildings and NLOS♯2 caused by mobile vehicles. (**b**) This figure shows the coordinate axis of the 3-axis MEMS sensors and the main lobe of the GNSS antenna when a pedestrian holds the smartphone. (**c**) The composite signal in the IQ diagram of PLL.

The multipath effect has different impacts on different GNSS measurements, such as pseudorange, carrier phase, and CN_0_R. The pseudorange multipath error is particularly important for the final positioning accuracy. Figure 2a shows the situation where a pedestrian encounters multipath, Figure 2b shows the orientation of the smartphone’s antenna, and Figure 2c shows the IQ diagram of multipath signal in Phase Lock Loop (PLL). The changes in NLOS signals compared with the LOS signal are mainly represented by three parameters, which are:

- α: the ratio of the signal amplitude of NLOS to that of LOS,
- ψ: the phase shift of NLOS with respect to LOS,
- δ: an additional path length of NLOS with respect to LOS.

As an example, Figure 2c shows the IQ diagram of the composite signal, composed of LOS and two NLOS signals in PLL; the amplitude of the LOS signal is $A_{0}$, the amplitude of NLOS♯1 is $A_{1}=\alpha_{1}A_{0}$ and the phase shift is $\psi_{1}$; the amplitude of NLOS♯2 is $A_{2}=\alpha_{2}A_{0}$ and the phase shift is $\psi_{2}$. If the number of NLOS signals is *n*, the pseudorange multipath error τ can be expressed using the above three parameters as [31]:

(2) $$\tau =\frac{\sum_{i=1}^{n}\alpha_{i}\delta_{i}\cos\psi_{i}}{1+\sum_{i=1}^{n}\alpha_{i}\cos\psi_{i}}$$

Besides, CN_0_R affected by NLOS can be expressed as [31]:

(3) $${\mathrm{CN}}_{0}R=\frac{2BW_{n}}{f_{s}T_{i}}A_{0}\left[1+\sum_{i=1}^{n}\alpha_{i}^{2}+\sum_{i=1}^{n}\alpha_{i}\cos\psi_{i}\right]$$

where $BW_{n}$ is the noise bandwidth of the receiver, $f_{s}$ is the A/D sampling frequency, $T_{i}$ is the integral period, and $A_{0}$ is the amplitude of the LOS signal.

## 4. Pedestrian Dead Reckoning

### 4.1. Step Detection

When a pedestrian walks, the output of the accelerometer is a periodic signal or a regular pattern. Therefore, scholars exploit this property to determine whether a pedestrian is taking a step. Traditional step detection methods include zero-crossing detection [38], peak detection [39], auto-correlation detection [40], and spectrum analysis [41], etc. In this paper, we adopt the dynamic threshold zero-crossing detection method [42]. This method uses a low-pass filter with a cut-off frequency of 2.1 Hz to preprocess the raw data of the accelerometer to reduce noise, and then uses a finite state machine to control the state transition between walking and non-walking, which can achieve an accuracy of 98%.

### 4.2. Stride Length Estimation

After the pedestrian step is detected, there are several models [43,44,45] to estimate the stride length, such as the empirical model, linear model, non-linear model, and machine learning model. The stride length model used here is a binary linear regression model, and the expression is as follows [45]:

(4) $$\mu =\beta_{1}\nu_{s}+\beta_{2}\sigma_{a}^{2}+\beta_{3}$$

where $\nu_{s}$ is the stride frequency and $\sigma_{a}^{2}$ is the acceleration variance. Parameters $\beta_{1}$, $\beta_{2}$, and $\beta_{3}$ need to be determined using off-line training. Once determined, these parameters can be used to estimate the stride lengths of different pedestrians in real-time applications.

### 4.3. Heading Estimation

Heading estimation is the most important step in PDR because the heading estimation error will accumulate, resulting in a severe distortion between the estimated trajectory and the groundtruth. The heading estimation usually uses the data from a gyroscope, magnetometer, or both of them [46,47]. Since the gyroscope generates a cumulative error during integration, and the magnetometer is susceptible to interference from the surrounding magnetic field, these two have obvious complementary characteristics. In this paper, we combine these two different sensors to estimate the optimal heading [48].

## 5. GNSS Real-Time Difference

### 5.1. GNSS Pseudorange Double-Difference

GNSS raw measurements include pseudorange, carrier phase, and CN_0_R, etc. Among them, pseudorange *P* and carrier phase Φ are expressed as follows:

(5) $$P=\rho +c\left(\delta t_{u}-\delta t^{s}\right)+I+T+\tau +\epsilon_{P}$$

(6) $$\Phi =\lambda^{-1}\left[\rho +c\left(\delta t_{u}-\delta t^{s}\right)-I+T+\phi \right]+Z+\epsilon_{\Phi }$$

where ρ represents the real distance, $\delta t_{u}$ and $\delta t^{s}$ represent the clock bias of the user’s smartphone and satellite, respectively, *c* is the speed of light in a vacuum, λ is the wavelength of the carrier, *I* and *T* represent ionospheric and tropospheric delay, respectively, *Z* is the integer ambiguity of the carrier phase, τ and ϕ represent the multipath error of pseudorange and carrier phase, respectively, and ϵ represents the sum of other errors dominated by the thermal noise. The double-difference pseudorange is defined as:

(7) $$\begin{array}{cc} \nabla \Delta P_{ur}^{ij} & =\left(P_{u}^{i}-P_{r}^{i}\right)-\left(P_{u}^{j}-P_{r}^{j}\right)\\ & =\nabla \Delta \rho_{ur}^{ij}+\nabla \Delta \tau_{ur}^{ij}+\nabla \Delta \epsilon_{P} \end{array}$$

where ∇Δ is the double-difference operator. The Earth-centered, Earth-fixed (ECEF) coordinates of the reference station *r* are known, while the location of the user *u* is unknown. Superscripts *i* and *j* denote the ith and jth satellites, respectively, where the jth satellite is the reference satellite, and we select the satellite with the maximum elevation as the reference. Note that RTD is a short-baseline double-difference model, so *I* and *T* in Equation (5) and Equation (6) are considered common mode errors when constructing the double-difference, and therefore are eliminated. Comparing Equation (5) and Equation (7), we can see that the double-difference pseudorange has a more concise expression than the un-difference pseudorange. In Equation (7), the residual errors are mainly multipath error $\nabla \Delta \tau_{ur}^{ij}$ and thermal noise $\nabla \Delta \epsilon_{P}$.

### 5.2. Double-Difference Code-Minus-Carrier

Similar to constructing the double-difference pseudorange using Equation (5) and Equation (7), we use Equation (6) to construct the double-difference carrier phase, as follows:

(8) $$\nabla \Delta \Phi_{ur}^{ij}=\lambda^{-1}\nabla \Delta \rho_{ur}^{ij}+\nabla \Delta Z_{ur}^{ij}+\nabla \Delta \epsilon_{\Phi }$$

From above, we can see that the double-difference carrier phase mainly includes unknown integer ambiguity $\nabla \Delta Z_{ur}^{ij}$ and thermal noise $\nabla \Delta \epsilon_{\Phi }$, and because the integer ambiguity of the smartphone has cycle slips and is difficult to be fixed, it is infeasible to implement Real-Time Kinematic (RTK). Since the multipath error ϕ in Equation (6) is very small, generally, at the centimeter level, it has little effect on improving the RTD solution, so we ignore this term in Equation (8).

According to Equations (7) and (8), we introduce a combined observation called DDCMC, as follows [49]:

(9) $$\begin{array}{cc} \nabla \Delta \mathrm{CMC} & =\nabla \Delta P_{ur}^{ij}-\lambda \nabla \Delta \Phi_{ur}^{ij}\\ & =\nabla \Delta \tau_{ur}^{ij}-\lambda \nabla \Delta Z_{ur}^{ij}+\nabla \Delta \epsilon \end{array}$$

### 5.3. Multipath Partial Parameters Estimation

Since the parameters α, ψ, and δ in Equation (2) are difficult to obtain, it is not possible to calculate the multipath error directly. However, we find that Equation (3) contains α and ψ, but it only lacks δ in Equation (2). Because CN_0_R is a direct output and the pseudorange observations contain multiple errors including multipath error, we propose a parameter estimation method called MPPE, which uses CN_0_R to estimate the multipath parameters α and ψ. Suppose that within a very short time window, α is a constant and remains unchanged, while ψ and δ change with time. Compared with ψ and δ, the change of α is not particularly important for multipath estimation and can be simplified. The time-varying expression of ψ can be modeled as follows:

(10) $$\psi_{i}\left(t\right)=\omega_{i}t+\gamma_{i}$$

where $\omega_{i}$ is the angular frequency and $\gamma_{i}$ is the initial phase.

If we use *K* to replace the constant term $\frac{2BW_{n}}{f_{s}T_{i}}A_{0}$ in Equation (3) and substitute Equation (10) into Equation (3), then we have:

(11) $${\mathrm{CN}}_{0}R\left(t\right)=K\left[1+\sum_{i=1}^{n}\alpha_{i}^{2}+\sum_{i=1}^{n}\alpha_{i}\cos\left(\omega_{i}t+\gamma_{i}\right)\right]$$

For the CN_0_R time sequence in Equation (11), we apply FFT to it and transform it to the angular frequency domain. Then, the amplitude of the zero frequency component is extracted and recorded as $\hat{A_{0}}$, and all other peaks in the spectrum are sorted in descending order of amplitude. The largest *n* peak amplitude values $\hat{A_{i}}$ with corresponding angular frequency $\hat{\omega_{i}}$ and phase $\hat{\gamma_{i}}$ are selected; in order to avoid noise, the choice of *n* is related to a threshold, and the threshold requires that the sum of the energy of the selected peaks should exceed 95% of the total non-zero frequency energy.

Finally, we can estimate the time-varying parameters α and ψ(t) using the above known quantities. The detailed derivation of MPPE is provided in Appendix A.

### 5.4. DDCMC Filter Design

It should be noted that Equation (2) is the expression of the un-difference pseudorange multipath error, but we need to deal with the double-difference form in RTD. Let us review Equation (7); it can be expanded as follows:

(12) $$\nabla \Delta \tau_{ur}^{ij}=\left(\tau_{u}^{i}-\tau_{r}^{i}\right)-\left(\tau_{u}^{j}-\tau_{r}^{j}\right)\approx \tau_{u}^{i}-\tau_{u}^{j}$$

In practice, the reference station is usually built in an environment without interference, such as setting the antenna at a high platform or adopting a survey-grade choke ring antenna, which can basically avoid the impact of multipath on the observation of the reference station. Therefore, there are $\tau_{r}^{i}\approx 0$ and $\tau_{r}^{j}\approx 0$ in Equation (12). In other words, only the multipath errors of the user’s smartphone need to be considered.

MPPE is able to estimate two parameters of the pseudorange multipath error, which are α and ψ. However, δ in Equation (2) cannot be obtained from the CN_0_R sequence. Therefore, we also need to estimate δ, and thus, obtain the estimation of the double-difference pseudorange multipath error in Equation (12).

From Equation (A4), we obtain $\kappa_{i}\left(t\right)$ corresponding to the $i^{th}$ NLOS, so that the vector κ consisting of *n* NLOS is as follows:

(13) $$\kappa ={\left[\begin{array}{cccc} \kappa_{1} & \kappa_{2} & \cdots & \kappa_{n} \end{array}\right]}^{⊤}$$

Similarly, the unknown vector δ of *n* NLOS is as follows:

(14) $$\delta ={\left[\begin{array}{cccc} \delta_{1} & \delta_{2} & \cdots & \delta_{n} \end{array}\right]}^{⊤}$$

Considering the relationship between Equation (2), Equation (9), and Equation (12), we design a KF called the DDCMC filter to estimate the unknown δ corresponding to $\tau_{u}^{i}$ and $\tau_{u}^{j}$, denoted by $\delta_{u,i}$ and $\delta_{u,j}$, respectively. The observed quantities of the DDCMC filter are ∇ΔCMC, while the state quantities are $\delta_{u,i}$ and $\delta_{u,j}$, as well as the unknown double-difference integer ambiguities $\nabla \Delta Z_{ur}^{ij}$. The design details of the DDCMC filter are shown in Appendix B, and we note that its state transfer is required for the control input; see Equation (A8). Finally, we can obtain the estimation of the pseudorange double-difference multipath error by substituting the outputs $\delta_{u,i}$ and $\delta_{u,j}$ of the DDCMC filter into the following:

(15) $$\nabla \Delta \tau_{ur}^{ij}=\kappa_{u,i}^{⊤}\delta_{u,i}-\kappa_{u,j}^{⊤}\delta_{u,j}$$

where $\kappa_{u,i}$ and $\kappa_{u,j}$ correspond to $\tau_{u}^{i}$ and $\tau_{u}^{j}$, respectively.

## 6. Methodology of GNSS/PDR Fusion

### 6.1. GNSS Dead Reckoning

By solving the pseudorange double-difference equation constructed by $\nabla \Delta P_{ur}^{ij}$, we obtain the baseline solution b in the ECEF frame. Assuming that the ECEF coordinate of the reference station is r, then r+b is the real-time ECEF coordinate of the user smartphone. The ECEF coordinates can be easily converted to Latitude-Longitude-Height (LLH) or East-North-Up (ENU) coordinates. Because PDR only considers 2D coordinates, the ECEF coordinates output by RTD need to be converted to ENU coordinates. In order to unify the GNSS outputs (ENU coordinates) and the PDR outputs (stride length and heading), we convert GNSS outputs into dead reckoning mode and define the GNSS heading and stride length. Assuming that $\left(E_{k},N_{k},U_{k}\right)$ and $\left(E_{k+1},N_{k+1},U_{k+1}\right)$ represent the ENU coordinates of two adjacent epochs *k* and k+1, then the GNSS stride length is calculated as:

(16) $$\mu_{k+1}=\sqrt{{\left(E_{k+1}-E_{k}\right)}^{2}+{\left(N_{k+1}-N_{k}\right)}^{2}}$$

and the GNSS heading is calculated as:

(17) $$\theta_{k+1}=\arctan\left(\frac{E_{k+1}-E_{k}}{N_{k+1}-N_{k}}\right)$$

### 6.2. PDR Output Synchronization

PDR updates its coordinates only when it detects a step, so that PDR outputs are burst and unpredictable. However, GNSS outputs the position solution at a certain frequency; for example, the highest update rate of GNSS observations in smartphones is 1 Hz. In order to fuse these asynchronous data in real-time, it is necessary to make the update frequency of the two systems consistent.

**Figure 3:**
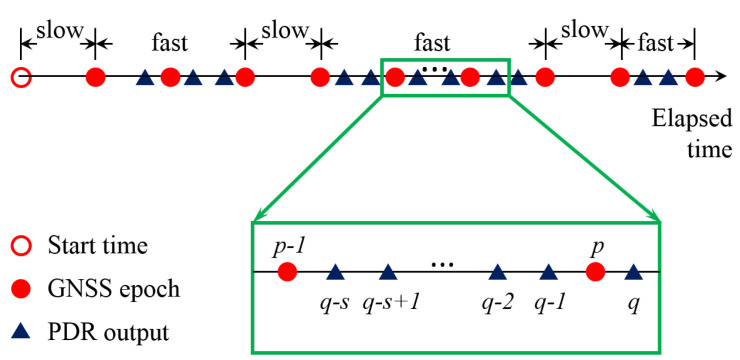
Schematic diagram of PDR and GNSS output synchronization.

Figure 3 shows a schematic diagram of data synchronization, and the basic idea of data synchronization is to merge the burst PDR outputs into periodic outputs. According to the characteristics of pedestrian walking, there are two cases in one GNSS interval: (1) no PDR output; (2) at least one PDR output. The former is called a slow step, and the latter is called a fast step. When a person walks normally, he can usually complete at least one step within 1 s. If he cannot, it is more likely that he does not make a decision for the next step. In general, people take fast steps when they are walking continuously, and slow steps happen occasionally. Note that most fast steps have only one or two PDR outputs within 1 s. In addition, if there are no steps for a long time, we call this case a stop. As shown in Figure 3, there are *s* PDR outputs between GNSS epoch p−1 and *p*, and *q* is the first PDR output that exceeds GNSS epoch *p*. Then, we can calculate the PDR stride length at GNSS time *p* as follows:

(18) $$\mu_{p}=\sqrt{{\left(\sum_{i=0}^{s-1}\mu_{q-i}\sin\theta_{q-i}\right)}^{2}+{\left(\sum_{i=0}^{s-1}\mu_{q-i}\cos\theta_{q-i}\right)}^{2}}$$

and the PDR heading at GNSS time *p* is calculated as follows:

(19) $$\theta_{p}=\frac{1}{s}\sum_{i=0}^{s-1}\theta_{q-i}$$

### 6.3. Joint Stride Length and Heading Estimation

Traditional GNSS/PDR fusion algorithms are usually based on KF or EKF, but these algorithms do not make good use of the difference between the GNSS and PDR errors. They simply feed the outputs of the two subsystems into the filter in the same way; therefore, the final filtering results often retain some characteristics of the GNSS or PDR errors. The fusion algorithm proposed in this paper also needs to design a unique KF to complete the filtering fusion, but before sending the data into KF, we first implement the joint stride length estimation and the joint heading estimation, respectively.

Joint heading estimation:

(20) $$\left\{\begin{array}{cc} \theta_{k+1}^{\mathrm{JOINT}} & =\frac{\Theta_{k+1}}{M}+\frac{M-1}{M}\left(\theta_{k}^{\mathrm{JOINT}}+\theta_{k+1}^{\mathrm{PDR}}-\theta_{k}^{\mathrm{PDR}}\right)\\ \Theta_{k+1} & =w_{1}\theta_{k+1}^{\mathrm{GNSS}}+\left(1-w_{1}\right)\theta_{k+1}^{\mathrm{PDR}} \end{array}\right.$$

where *M* is the smoothing coefficient and $w_{1}$ is a weighting factor. *M* should not be set to be too large or too small; if it is too large, the tracking of the heading becomes dull, and if it is too small, the filtering effect is not good, so it is usually set to 30 to 50. $w_{1}$ usually takes a value of from 0.6 to 0.9. Equation (20) is an iterative equation and it is designed to use a PDR heading with low noise power to smooth the GNSS heading with high noise power, and only the difference of PDR headings is used, so the cumulative error in the final joint heading estimation is also suppressed.

Joint stride length estimation:

(21) $$\mu_{k+1}^{\mathrm{JOINT}}=w_{2}\mu_{k+1}^{\mathrm{GNSS}}+\left(1-w_{2}\right)\mu_{k+1}^{\mathrm{PDR}}$$

where $w_{2}$ is a weighting factor. According to the test results later, we find that the stride length accuracy of PDR estimation is better than that of GNSS estimation. Therefore, Equation (21) is a simple weighting formula, and the joint stride length is more dependent on the PDR, so $w_{2}$ is generally set to 0.1.

### 6.4. Fusion Filtering and Trajectory Updating

After the joint stride length and heading estimation, a KF is applied to filter out the remaining noise. The observations of this KF are $\theta_{k+1}^{\mathrm{JOINT}}$ and $\mu_{k+1}^{\mathrm{JOINT}}$, and the states of this KF can be selected as $\left(x,y\right)$ or $\left(\mu ,\theta \right)$, etc. Because it is difficult to predict pedestrian behavior, the state transition is generally considered to be the state of the previous moment, or by using other methods to predict behavior. For the KF design of PDR, please refer to [50,51]. Finally, the filtering results of KF are sent to the fusion trajectory update module, and the pedestrian position is calculated according to Equation (1).

## 7. Experiments and Results

### 7.1. Experimental Setup

In this paper, we choose Xiaomi 8 UD as the test smartphone, whose GNSS chipset is Broadcom BCM47755, the 6-axis MEMS IMU is Qualcomm ICM20690, and the 3-axis magnetometer is akm ak0991x. BCM47755 supports both the GPS L1 and L5 dual-frequency signals, but only the GPS L1 signals have been used in our experiments. We develop a data collection APP using the Android APIs, and use our own post-processing code on the computer to process and analyze the data, to verify the effectiveness of our proposed method. By turning off the duty-cycle option, we can track the continuous GNSS carrier. In addition, we adopt the Huace B5 survey-grade GNSS receiver as the reference station, and use it to collect the static data. We also use the Huace i70II receiver and CORS to measure some positions with RTK, the accuracy of which is centimeter-level.

In order to evaluate the performance of our proposed method, we designed three tests, which are: (1) The control point test is used to analyze the error characteristics of a standalone system (Only PDR or Only RTD); (2) The static multipath test is used to analyze the suppression effect of the DDCMC filter on the pseudorange multipath error of the smartphone; (3) The GNSS/PDR fusion test is used to analyze the positioning accuracy of different standalone systems and fusion systems.

**Figure 4:**
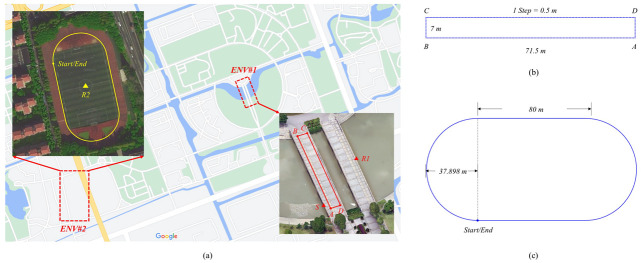
Experimental environments. (**a**) Two test sites. (**b**) The track used for control point test. (**c**) The track used for GNSS/PDR fusion test.

- Control point test: See Figure 4a,b; the trajectory of our selected control point test is a rectangle (see ENV♯1). There are markers on the ground, and the distance between the two markers is 0.5 m, which also equals the stride length of each step. The long side of the rectangle has 143 steps, and the short side has 14 steps. The four corner points (A, B, C, and D) of the rectangle are measured using our RTK devices, so that we can obtain the groundtruth of the heading for each edge. The reference station is set at R1.
- Static multipath test: See Figure 4a; this test constructs a pair of double-difference observations, where the reference station is located at R1 and the test smartphone is located at S. The groundtruth of these two locations are measured in advance using RTK. R1 is located in the middle of the bridge, away from the reflector, so that the reference station is almost unaffected by the multipath. S is near the surrounding obstacles, such as the trees in the south, constituting a multipath reflector, and so the test smartphone receives a more severe multipath impact.
- GNSS/PDR fusion test: See Figure 4a,c; the trajectory of our selected fusion test is a standard 400 m track (see ENV♯2). The reference station is set at R2.

### 7.2. Control Point Test

**Figure 5:**
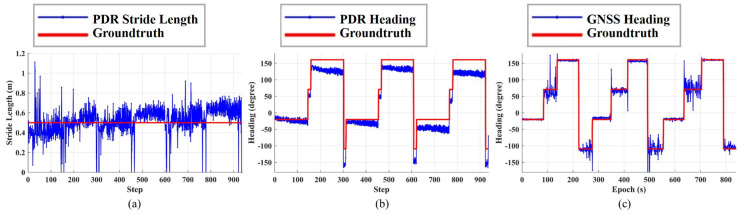
Results of control point test. (**a**) PDR stride length estimation. (**b**) PDR heading estimation. (**c**) GNSS heading estimation.

The tester walks three laps along the track, and the route of one lap is A-B-C-D-A. The number of steps in one lap is 314, so the total number of steps is 942, and the whole test time is about 15 min. At each corner point, the tester stands still for approximately 30 s and completes a turn-in-place. The results are shown in Figure 5 and Table 1.

Figure 5a,b respectively show the estimated stride length and heading of standalone PDR, in which the red line is the groundtruth and the blue line is the estimated results. We can see the periodic pattern, which corresponds to the repeated results of three laps. The total number of the estimated steps is 937, so the success rate of the step detection is 99.5%. After analysis, the main reason for some detection errors is that the step detection module merges some fast steps into one step.

As we can see from Figure 5a, the stride length estimation of PDR is basically around the groundtruth, but there are two unusual findings. One is that there are outliers with large stride length estimation errors; for example, some steps are estimated to be more than 1 m, while some steps are zero. After analysis, we find that these outliers mainly appear at the moment when the tester is about to stop or walk. Another is that the error of the stride length estimation increases with time. When the tester walks continuously, the error of stride length estimation is usually on the centimeter level. From Table 1, the average stride length estimation errors of each lap are −0.02 m, 0.04 m, and 0.09 m. It can be seen that the stride length error of the third lap is 11 cm larger than that of the first lap.

The comparison between the heading estimation and the groundtruth of PDR is shown in Figure 5b. We can see that the heading estimation is more accurate in the initial stage, and that the error of the heading estimation gradually increases over time. Table 1 shows the average heading estimation error of three laps, which are −16.5 °, −18.7°, and −33.5°, respectively. These heading errors are too large, which eventually leads to complete trajectory distortion.

Figure 5c shows the heading estimation error of GNSS. Here, GNSS adopts RTD positioning without multipath mitigation. Because RTD is a fixed frequency output, the GNSS stride length is not shown here because it is meaningless and only meaningful during fusion. The average heading estimation errors of the three laps are shown in Table 1, which are −2.9°, −0.9°, and 2.2°, respectively. It can be seen that there is no large drift in GNSS headings; however, there are many outliers with the large errors shown in Figure 5c, indicating that the GNSS results are susceptible to interference, mainly multipath interference.

### 7.3. Static Multipath Test

In this test, the test smartphone is placed at S and the reference station is placed at R1. They record the static data at the same time, including observation and ephemeris. We verify the performance of the proposed multipath mitigation by observing a double-difference pair composed of GPS G10 and GPS G32. Since the CN_0_R of G32 is always better during the whole observation time, G32 is selected as the reference satellite. The total test time is about 30 min.

**Figure 6:**
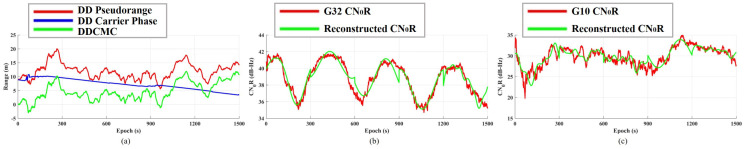
Results of the static multipath test. (**a**) Double-difference observations. (**b**) Reconstructed CN_0_R of G32 via MPPE. (**c**) Reconstructed CN_0_R of G10 via MPPE.

Figure 6a shows the double-difference pseudorange, double-difference carrier phase, and DDCMC. Comparing Equations (7)–(9), we can see that there is no $\nabla \Delta \rho_{ur}^{ij}$ in DDCMC, so we can use DDCMC to separate the double-difference pseudorange multipath error and the double-difference integer ambiguity directly. Figure 6b,c shows the reconstructed CN_0_R of G10 and G32 using MPPE, respectively. The reconstructed CN_0_R extracts the principal components of the raw CN_0_R and ignores the high-frequency noise components. In addition, the fluctuation of the CN_0_R waveform reflects the severe multipath effect in the current satellite observations.

**Figure 7:**
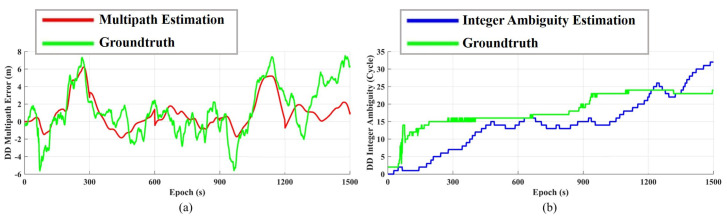
Outputs of DDCMC filter. (**a**) Multipath error estimation. (**b**) Integer ambiguity estimation.

Figure 7 shows the comparison between the outputs of the DDCMC filter and groundtruth, where Figure 7a is the double-difference pseudorange multipath estimation and Figure 7b is the double-difference integer ambiguity. It is necessary to explain the acquisition approach of the groundtruth, because the coordinates of R1 and S are known, and the coordinates of the satellites are calculated via ephemeris, so $\nabla \Delta \rho_{ur}^{ij}$ in Equations (7) and (8) is also known, and then the thermal noise term is removed via fine filtering, and so the rest terms are the groundtruth of the double-difference pseudorange multipath and integer ambiguity.

**Figure 8:**
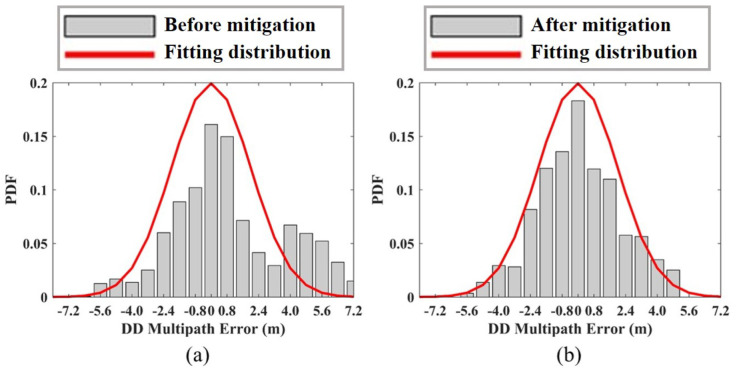
Probability density function of double-difference multipath error. (**a**) Before multipath mitigation. (**b**) After multipath mitigation.

From Figure 7a, we can see that the proposed method is able to well estimate the large offset in the multipath error, such as the range of (200, 300) and (1000, 1200). Figure 8 shows the result of multipath mitigation from the other side, in which Figure 8a is the PDF of measurement errors before multipath mitigation, and Figure 8b is the PDF of measurement errors after multipath mitigation. It can be seen that the PDF of multipath errors is usually not Gaussian distribution; for example, errors that are larger than 4 m in Figure 8a are outside of the Gaussian distribution fitting curve. After mitigation, the error PDF can better fit the Gaussian distribution curve, and the energy of residuals that are larger than 4 m is more concentrated near the expectation, so that the remaining error can be regarded as the thermal noise of Gaussian distribution, and KF can be used for optimal filtering.

Another outcome of the DDCMC filter is shown in Figure 7b; that is, double-difference integer ambiguity. It does not directly affect multipath mitigation, but it reflects many facts. By observing the groundtruth, as shown by the green line, we can see that the groundtruth is not an ideal constant, but a stepped waveform. At present, the carrier tracking of the smartphone-grade GNSS chipset is still imperfect, so we cannot apply RTK directly. The estimated integer ambiguity is shown by the blue line; although there is a large difference between the estimation and the groundtruth, it also basically tracks the change of the groundtruth. In fact, it is more difficult to solve the integer ambiguity than multipath estimation through smartphone-grade observations; therefore, RTD with multipath mitigation is more practical than RTK, based on the performance of the current ultra-low-cost mass-market GNSS chipset.

### 7.4. GNSS/PDR Fusion Test

**Figure 9:**
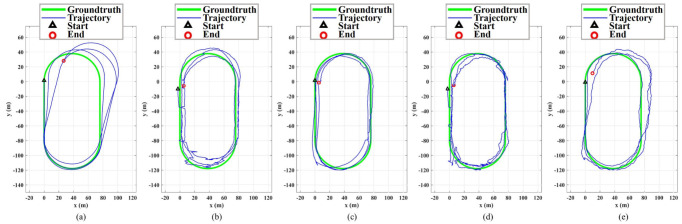
Results of three-lap GNSS/PDR fusion test of five pedestrian positioning methods. (**a**) Only PDR trajectory. (**b**) Only RTD trajectory. (**c**) The trajectory of the proposed method. (**d**) MREKF trajectory. (**e**) ESC trajectory.

**Figure 10:**
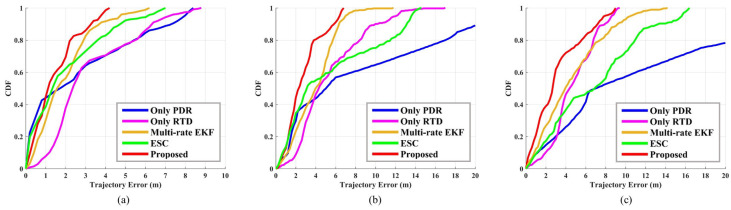
Cumulative distribution function of three-lap GNSS/PDR fusion test of five pedestrian positioning methods. (**a**) CDF of the first lap. (**b**) CDF of the second lap. (**c**) CDF of the third lap.

The tester holds the smartphone in his right hand and walks counterclockwise for three laps along the standard 400 m track. The start and end points are shown in Figure 4c, and the total test time is about 20 min. At the same time, we set the reference station at R2 and start the static recording. The results of this test are shown in Figure 9 and Figure 10, and in Table 2. Five methods are tested, which can be divided into two categories: one is standalone systems, including Only PDR and Only RTD; and the other is fusion systems, including our proposed algorithm, MREKF, and ESC.

Figure 9a shows the trajectory of PDR after three laps. The initial position and heading of PDR are given by the priori true values. During the test, a total of 1938 steps are detected, and the trajectory of the first lap is very close to the groundtruth; however, due to the cumulative error in the heading estimation, the next two laps deviate far from the groundtruth. Figure 9b shows the trajectory of RTD after three laps, where multipath mitigation is not applied, and so multipath errors are reflected in the trajectory. For example, the positioning errors at the two arc segments are much larger compared to the straight segments; this is because some satellites are partially blocked by the orientation of the tester at that time. In addition, the pseudorange observations of RTD are smoothed by using the carrier phase observations, so the thermal noise has been reduced and the final trajectory is smoother compared to the single-point solutions. Compared with the first lap, the third lap does not have significant drift.

Figure 9c shows the trajectory of the proposed method; the RMSEs corresponding to three laps are 1.63 m, 3.19 m, and 4.71 m, respectively. Figure 9d shows the trajectory of MREKF; the RMSE corresponding to the three laps are 2.37 m, 3.02 m, and 4.81 m, respectively. Figure 9e shows the trajectory of ESC, the RMSE corresponding to three laps are 2.70 m, 7.15 m, and 8.21 m, respectively. Compared with the two standalone systems, the three fusion methods are improved in different aspects. MREKF relies more on GNSS, so the trajectory is distorted due to the multipath errors at two arc segments, while ESC relies more on PDR, so it also drifts on the second and third laps. Meanwhile, our fusion system avoids these negative impacts, and it can run well for a long time.

Figure 10 shows the CDF of five methods. In order to show the comparison of long-time positioning performance, we present the results of each lap and put them in the same picture. In the comparison of the first lap, as shown in Figure 10a, our proposed method has the best performance, except when the CDF is less than 0.5, PDR works best here because we provide it with very accurate initial values. The results of the first lap from best to worst are the proposed method, MREKF, ESC, Only PDR, and Only RTD, respectively. As shown in Figure 10b, on the second lap, the performances of ESC and PDR are severely degraded because of the rapid growth of heading drift. The results of the second lap from best to worst are the proposed method, MREKF, Only RTD, ESC, and Only PDR, respectively. As shown in Figure 10c, on the last lap, our proposed method is still better than the other methods, and the performances of MREKF and ESC are even worse than that of RTD. After analysis, the performance of the MEMS MARG on the third lap has a greater impact on the fusion system after a long time, which leads to significant performance degradation. The results of the third lap from best to worst are the proposed method, Only RTD, MREKF, ESC, and Only PDR, respectively.

## 8. Conclusions

In this paper, a multi-phase GNSS/PDR fusion framework based on ultra-low-cost mass-market GNSS and MEMS MARG is proposed. Firstly, in order to achieve the data fusion of two heterogeneous positioning systems, several engineering innovations have been implemented, such as GNSS coordinate conversion, data synchronization, and the application of RTD instead of traditional single-point positioning. Next, we illustrate and analyze the main errors in the fusion system, including GNSS multipath errors and MEMS cumulative errors, which are relevant to the application scenario of pedestrian positioning and the performance of the ultra-low-cost sensors. For multipath errors, we propose the CN_0_R-based MPPE and the design principle of the DDCMC filter, and thus, we demonstrate the feasibility of multipath mitigation based on smartphone-grade low-quality observations; the experimental result shows that the proposed method can effectively mitigate the double-difference multipath error exceeding 4 m. Finally, we propose a novel fusion algorithm for RTD and PDR, including joint stride length estimation, joint heading estimation, and filter design, using the joint estimation before fusion filtering to minimize the cumulative errors of PDR. The proposed fusion method achieves a minimum error RMSE of 1.63 m in positioning accuracy and a minimum error RMSE of 4.71 m in long-time robustness for 20 min of continuous walking in the comparative tests. CEP is also used to quantitatively analyze the uncertainty of fusion positioning errors here. Specifically, CEP50% of the proposed method is 1.12 m, 2.29 m, and 2.67 m; and CEP90% is 3.27 m, 5.83 m, and 7.19 m, corresponding to three laps of testing, respectively. These results demonstrate that the proposed method is also superior to other fusion methods in terms of CEP indicators.

## Figures and Tables

**Table 1:** Results of three-lap control point test for two standalone methods.

Evaluation Index	Lap 1	Lap 2	Lap 3
Detected steps	311	309	316
Detection success rate (%)	99.1	98.4	99.4
PDR stride length error (m)	−0.02	0.04	0.09
PDR heading error (°)	−16.5	−18.7	−33.5
GNSS heading error (°)	−2.9	−0.9	2.2

**Table 2:** Comparison of three-lap GNSS/PDR fusion test of five pedestrian positioning methods.

Methods	RMSE (m)			CEP50% (m)			CEP90% (m)		
	Lap 1	Lap 2	Lap 3	Lap 1	Lap 2	Lap 3	Lap 1	Lap 2	Lap 3
Only PDR	3.76	10.99	14.42	1.71	5.02	7.00	7.31	20.80	27.40
Only RTD	3.77	6.23	5.21	2.35	4.30	4.37	6.34	9.98	7.91
MREKF	2.37	**3.02**	4.81	1.57	3.99	3.94	3.69	6.33	9.19
ESC	2.70	7.15	8.21	1.34	3.10	6.88	4.73	13.13	13.28
Ours	**1.63**	3.19	**4.71**	**1.12**	**2.29**	**2.67**	**3.27**	**5.83**	**7.19**

## Data Availability

Not applicable.

## Appendix A

After determining $\hat{A_{0}}$, $\hat{A_{i}}$, $\hat{\omega_{i}}$, and $\hat{\gamma_{i}}$ (i=1,2,…,n), we can reconstruct the CN_0_R as follows:

(A1) $$\hat{{\mathrm{CN}}_{0}R}\left(t\right)\approx \hat{A_{0}}+\sum_{i=1}^{n}{\hat{A}}_{i}\cos\left(\hat{\omega_{i}}t+\hat{\gamma_{i}}\right)$$

Comparing Equation (11) and Equation (A1), the following equations can be listed to solve the unknown parameters in Equation (11), they are:

(A2) $$\left\{\begin{array}{cc} \begin{array}{lll} {\hat{A}}_{0} & = & K\left(1+\sum_{i=1}^{n}\alpha_{i}^{2}\right)\\ {\hat{A}}_{i} & = & K\alpha_{i}\\ {\hat{\omega }}_{i} & = & \omega_{i}\\ {\hat{\gamma }}_{i} & = & \gamma_{i} \end{array}, & \forall i=1,2,\ldots ,n \end{array}\right.$$

From Equation (11), the quantities to be solved are *K*, $\alpha_{i}$, $\omega_{i}$, and $\gamma_{i}$ (i=1,2,…,n). The latter two are obtained directly from Equation (A2), while *K* and $\alpha_{i}$ require solving of the first two equations in Equation (A2). Finally, we can obtain the following results:

(A3) $$\left\{\begin{array}{ccc} K & = & 0.5\left({\hat{A}}_{0}+\sqrt{{\hat{A}}_{0}^{2}-4\sum_{i=1}^{n}{\hat{A}}_{i}^{2}}\right)\\ \alpha_{i} & = & 2{\hat{A}}_{i}{\left({\hat{A}}_{0}+\sqrt{{\hat{A}}_{0}^{2}-4\sum_{i=1}^{n}{\hat{A}}_{i}^{2}}\right)}^{-1}\\ \psi_{i}\left(t\right) & = & {\hat{\omega }}_{i}t+{\hat{\gamma }}_{i} \end{array}\right.$$

According to $\alpha_{i}$ and $\psi_{i}\left(t\right)$ from Equation (A3), we construct an intermediate quantity κ, as follows:

(A4) $$\kappa_{i}\left(t\right)=\frac{\alpha_{i}\cos\psi_{i}\left(t\right)}{1+\sum_{j=1}^{n}\alpha_{j}\cos\psi_{j}\left(t\right)}$$

## Appendix B

The state x of the DDCMC filter contains the parameter δ that we need, and an additional output, the double-difference integer ambiguity. It has the following form:

(A5) $$x={\left[\begin{array}{ccc} \delta_{u,i}^{⊤} & \delta_{u,j}^{⊤} & \nabla \Delta Z_{ur}^{ij} \end{array}\right]}^{⊤}$$

The observation of the DDCMC filter is ∇ΔCMC. According to Equation (9), we have the following observation equation:

(A6) ∇ΔCMC=Hx+∇Δϵ

(A7) $$H=\left[\begin{array}{ccc} \kappa_{u,i}^{⊤} & -\kappa_{u,j}^{⊤} & -\lambda \end{array}\right]$$

where H is the design matrix; $\kappa_{u,i}$ and $\kappa_{u,j}$ are obtained from Equation (A4).

The state transfer of the DDCMC filter is determined by both the state value of the previous moment and the control input $u_{k}$, and the state transition equation is as follows:

(A8) $${\hat{x}}_{k|k-1}=x_{k-1|k-1}+u_{k}$$

where $u_{k}$ consists of the estimated angular frequency $\omega_{i}$ obtained in Equation (A2), the relationship between δ and ω is used to predict the state at the next moment, and the state $\nabla \Delta Z_{ur}^{ij}$ remains constant. Hence, it is expressed as follows:

(A9) $$u_{k}=\frac{\lambda }{2\pi }{\left[\begin{array}{ccc} \omega_{u,i}^{⊤} & -\omega_{u,j}^{⊤} & 0 \end{array}\right]}^{⊤}$$

(A10) $$\omega ={\left[\begin{array}{cccc} \omega_{1} & \omega_{2} & \cdots & \omega_{n} \end{array}\right]}^{⊤}$$

where $\omega_{u,i}$ and $\omega_{u,j}$ correspond to $\tau_{u}^{i}$ and $\tau_{u}^{j}$, respectively.
